# Evaluating frailty assessment tools and biological frailty markers in C57BL/6 female mice

**DOI:** 10.1007/s11357-024-01430-7

**Published:** 2024-12-03

**Authors:** Laís R. Perazza, Christopher M. Bougher, LaDora V. Thompson

**Affiliations:** Department of Physical Therapy, Boston University, Boston, MA USA

**Keywords:** Frailty Index, Physical Phenotype, Frailty assessment tools

## Abstract

Frailty is a complex condition characterized by a decline in multiple physiological systems, compromising an individual’s ability to maintain homeostasis. The onset and progression of frailty are linked to negative health outcomes such as disability, hospitalization, and mortality. To better understand frailty mechanisms, animal models have become valuable due to their accessibility to critical tissues and the ability to control variables. This study investigates frailty in female C57BL/6JN mice by comparing the two most widely accepted frailty assessment tools—the Physical Phenotype (PP) and the Frailty Index (FI). While FI relies on the accumulation of health deficits, PP focuses on functional physical performance. Our results suggest that these tools are complementary rather than interchangeable, each targeting distinct markers of frailty. To address the limitations of both, we propose a new combined assessment tool, the Vitality Phenotype (VP), which integrates physical performance and health deficits into a more comprehensive evaluation. We also explore the challenges in standardizing frailty cutoff values and the need for a reference group to enhance the accuracy of frailty identification. Lastly, we investigate the correlation between frailty criteria and biological markers, particularly bone mineral density (BMD), which shows a significant relationship with physical performance metrics such as grip strength and voluntary wheel activity, highlighting its potential role in frailty pathophysiology.

## Introduction

Frailty is a condition that arises from physiological systems deterioration to the point where resilience and repair mechanisms can no longer maintain homeostasis [1]. Understanding the progression of frailty is critical, as it is associated with poor health outcomes, including falls, incident disability, hospitalization, and mortality [2]. Over the past decades, various tools have been developed in both clinical and basic science research to identify the “multiple physiological systems decline” in frailty [3, 4]. However, disagreement among researchers regarding the best approach to assess frailty has led to the development of diverse assessment tools that focus on different clinical and physical characteristics of frailty [1, 5].

To date, two frailty assessment tools which utilize a variety of variables have become more accepted by the scientific community: the Physical Frailty Phenotype (PP) and the Frailty Index (FI) of Accumulative Deficits. The PP was developed by Fried and colleagues and relies on measuring dysregulation in physiological systems [2]. Individuals who perform poorly on at least three out of five predetermined phenotypic criteria—weakness, slowness, physical activity, exhaustion, and body weight loss—are classified as frail. In contrast, the Frailty Index, developed by Rockwood and colleagues relies on observing a number of predefined health deficits in a subject, with higher numbers of deficits corresponding to higher levels of frailty [6]. Over the past decade, these two assessment tools were reverse-translated to rodents [7, 8], significantly advancing frailty research. Using animal models allows researchers to access critical tissues and control lifestyle variables, enabling a more precise evaluation of frailty assessment tools. This approach also offers the ability to examine biological markers in tissue samples that would be difficult or impractical to obtain from human subjects, providing a deeper understanding of the mechanisms underlying frailty. Yet, despite mice physiological similarities to humans, defining the tools that best identify frailty in mice is rather challenging: age selection, cutoff definition, methods of physical evaluation, definition of scoring strategy, determining negative controls, sex differences, etc., are all variables that challenge standardization of frailty assessment tools. Herein, using a multi-project female cohort, we discuss differences in frailty status according to each of the most commonly used assessment tools (Frailty Index and Physical Phenotype) and discuss the outcome of merging both into a more comprehensive evaluation, the Vitality Phenotype. We further discuss the challenges of defining the cutoff values for each criterion and potential adaptations (considerations) to be applied to the scoring system [9].

Several theories considering multiple biological systems have defined frailty-related physiological impairments, while certain biological markers have been linked to frailty [10–13]. Yet, the heterogeneity of frailty assessment tools as well as the extensive variability across cohort phenotypes hinder the progress in the understanding of underlying pathophysiologies related to frailty onset. To better understand how some of the frailty-related biological markers associate with frailty criteria, we investigated associations between measurements of glycemia during oral glucose tolerance test (oGTT), inflammation, bone mineral density (BMD), and heart rate versus several positive frailty criteria.

## Methods

### Animals

One hundred ninety 17–27 month-old female C57BL/6JN mice were provided by the National Institute of Aging (NIA) rodent colony and were divided into age groups representing a spectrum from middle age (17–19 months old) to old (21–28 months old). Age distribution within the cohort is detailed in Tables 1 and 2. All protocols utilized in this study were approved by IACUC. The animal cohort constituted the sum of mice from multiple animal research projects in our laboratory over the past 3 years and a half, one of these studies was already published [14], and other manuscripts are in preparation.

**Table 1:** Age distribution in old cohort

Old age (mo)	Sample size distribution (total *n* = 190)
21	6
22–23	79
24	45
25	29
26	8
27	21
28	2

**Table 2:** Age distribution in cohort from studies that were run in parallel with a reference group (middle age)

Middle age (mo)	Sample size distribution (total *n* = 87)
17	40
18	17
19	30
Old age (mo)	Sample size distribution (total *n* = 127)
21	6
22	12
23	28
24	37
25	24
26	8
27	11
28	1

### Frailty assessment

Frailty in female mice was evaluated using three primary methods listed below. For each research project, assessments were conducted within the same diurnal period for all mice, with experimental groups alternating. Within each assessment, a single evaluator conducted all experiments.

#### Frailty Index (FI)

The FI was calculated based on a comprehensive list of health deficits, including rectal prolapse, coat condition, and other physical and behavioral characteristics, as previously described by Whitehead et al. [8]. Health deficits were assessed by the same blinded rater in each research project, and each deficit was scored as follows: 0 for no deficit, 0.5 for a mild deficit, and 1 for a severe deficit. Each mouse was assigned a FI score representing the proportion of deficits present out of the total assessed. Body weight and body temperature were measured weekly and analyzed separately in each research project (data not shown) and were, thus, excluded from the original version of the FI proposed by Whitehead et al. [8]. Also, the grip force test was included as a physical measure alone in Physical Phenotype and not merged together with the Index as originally described by Whitehead et al. [8].

#### Physical Phenotype (PP)

The PP was determined using a series of physical tests that measure functional performance, including walking speed, grip strength, endurance, physical activity, and body weight changes as previously described [14].

- Walking speed

Walking speed was measured using a rotarod (Pan-Lab Letica Rota-Rod L/S, Catalonia, Spain). Mice were placed on the rotarod at a constant speed of 4 rpm for 1 min to acclimate. After acclimation, the speed increased by 1 rpm every 8 s, reaching a maximum of 40 rpm over 5 min. The test continued until the mouse could no longer keep pace with the rotarod. Each mouse underwent three trials, with a 10-min rest period between trials, and the highest speed from the three trials was recorded as the walking speed.

- Strength

Grip strength was assessed using a grip strength meter (Grip Strength Test P/N 760483, Coulbourn Instruments, Whitehall PA), and maximal strength was recorded. Mice were gently lowered onto the grid to allow both front and hind paws to grip. The tail was pulled back steadily, keeping the torso horizontal by holding the tail’s base between the thumb and forefinger. When the mouse could no longer maintain its grip, the trial ended, and maximal grip strength was noted. Five trials were conducted for forelimb and hindlimb strength, with a 10-min rest between each trial. The highest and lowest results were discarded, and an average was calculated from the remaining three trials.

- Endurance

Endurance was measured on a six-lane motorized treadmill (Exer 3/6 Treadmill; Columbus Instruments, Columbus, OH). Mice started with a 5-min warm-up at 5 m/min on a level deck. After the warm-up, the speed increased by 1 m/min. Exhaustion was determined when the mouse required encouragement three consecutive times, and the distance covered in meters was recorded.

- Voluntary physical activity

Voluntary physical activity was tracked in individual activity cages equipped with a running wheel (Lafayette Instrument, IN, USA), with ad libitum access to food and water. The test included 1 day of acclimation followed by 4 experimental days. Total running distance was measured and recorded in kilometers per day using Scurry activity monitoring software (Lafayette Instrument, IN, USA). After each session, animals were observed for 20 min upon reintroduction to group housing to prevent injury from fighting.

#### Vitality Phenotype (VP)

In general, the VP consisted of combining the FI and the PP. Specifically, the health questionnaire used to generate the FI became a “health status marker” and was identified as a criterion (accumulation of health deficits) in the new VP. Thus, the VP includes endurance, grip strength, walking speed, physical activity, weight gain/loss, and accumulation of health deficits (6 criteria.) Briefly, the lowest 20th percentile for each frailty criterion corresponded to the cutoff value of that specific criterion. Animals whose performance would be equal to or lower than the cutoff for a given criterion would score positive for that criterion. Four or more positive frailty markers identified an animal as frail, two or three positive markers indicated prefrail, and one or zero specified robust.

### Biological markers

Several biological markers that have previously been associated with frailty in humans or mice were evaluated in the current study.

#### Metabolism

Glucose homeostasis was assessed by an oral glucose tolerance test that was performed as previously described [14]. In summary, glucose regulation was assessed after administering 1 g/kg body weight (BW) of a 50% dextrose solution via gavage in 6-h fasted animals. Blood samples were collected from the saphenous vein using a One Touch Ultra glucometer (LifeScan, Inc.) at 0, 15, 30, 60, 90, and 120 min post-gavage to monitor glycemic levels. Area under the curve (AUC) was calculated for each mouse to extract a single value that best represented the glycemic curve.

#### Bone mineral density (BMD)

Whole bone BMD was measured in the right tibia using a dual-energy X-ray absorptiometry (DEXA system, GE Lunar PixiMus, Madison, WI, USA). Tibia was collected at the time of euthanasia and fixed in 4% paraformaldehyde for later analysis. Samples were then placed in 1X phosphate-buffered saline (PBS) solution before scanning. Batches of 10–12 tibia samples were scanned to assess the whole BMD.

#### Inflammation

To determine the inflammatory status, monocyte chemoattractant protein-1 (MCP-1) was measured on plasma collected during cardiac puncture at euthanasia. A group of cytokines were measured using a multiplex immunology assay (MILLIPLEX® MAP Mouse Cytokine/Chemokine Magnetic Bead Panel, MilliporeSigma), but only MCP-1 was reported in the present study. Other cytokines were described in the original publication [14].

#### Cardiac function

Heart rate (HR) was determined by electrocardiography data (Fujifilm Visual Sonics Inc., Toronto, Ontario, Canada). Briefly, mice were anesthetized with isoflurane, and surface ECG signals (lead II via limb electrodes) were recorded using a 4-Channel Small Animal ECG/EMG Recorder with LabScribe software (IX-BIO4-SA, iWorx 2607 Systems, Inc.). The mice were placed in a supine position on a heated platform, with their paws connected to electrode needles. Body temperature was maintained between 35 and 38 °C. To keep the heart rate between 300 and 370 bpm, isoflurane was administered at 3% for induction and 1% for maintenance, with a flow rate of 0.4–0.8 l/min. After a 3-min acclimation period, ECG recordings were performed for a duration of 5 min.

### Statistical analysis

The correlations between frailty criteria and biological markers (AUC oGTT, BMD, MCP-1, HR) were analyzed using Pearson’s correlation coefficient. The strength of the correlation was expressed as the coefficient of determination (*R*^2^), with a two-tailed *P*-value calculated to assess statistical significance. The significance threshold was set at *P* ≤ 0.05. Multiple regression models were also employed to evaluate the combined effects of these criteria and biological markers, with the results interpreted in the context of the overall aging and vitality status of the animals. All statistical analyses were performed using GraphPad Prism Version 10.3.0.

## Results and discussion

### Frailty Index versus Physical Phenotype as complementary tools

There is a dearth of mice studies where frailty was determined by either one of the two major frailty assessment tools: the Frailty Index (FI) and the Physical Phenotype (PP). There have been multiple head-to-head comparisons of the Frailty Phenotype (FP) and Frailty Index (FI) in both clinical and preclinical settings. Clinical studies have highlighted key differences between these two tools in predicting outcomes like mortality, hospitalization, and functional decline [15–18]. Preclinical comparisons using murine models have similarly demonstrated differences in frailty outcomes, particularly depending on the assessment methodology, such as physical or lab-based measures [19, 20]. Seldeen and colleagues further emphasized the biological variability influencing these measures in female mice [21], reinforcing the importance of addressing these methodological differences when comparing the FP and FI in both clinical and preclinical research.

To date, it is not clear whether each tool would classify animals similarly. Determining which are the most relevant markers of frailty to be targeted by an assessment tool is a challenge, especially considering the multisystem nature of frailty-driven physiological decline. Still, the standardization of frailty classification across studies would greatly contribute to advances in the field of aging. Hence, one of the main objectives of this study was to determine the level of frailty of female C57BL/6 by comparing for the first time how both the mouse FI and the PP would classify female mice under the three main frailty categories: Robust, Prefrail, and Frail.

**Fig. 1:**
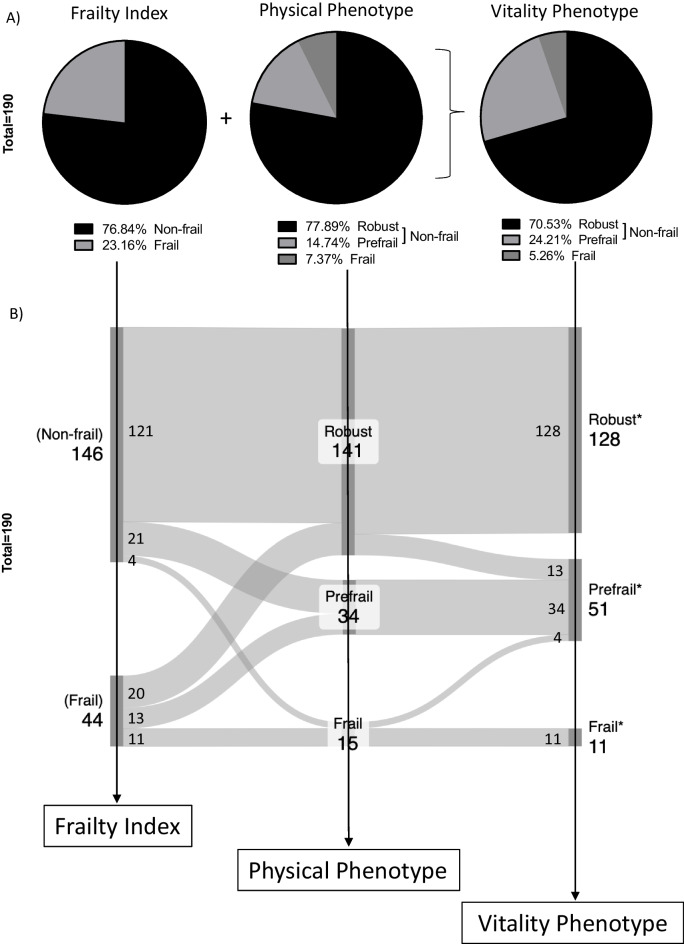
Frailty Index versus Physical Phenotype as complementary tools. Comparison of frailty classification using Frailty Index (FI), Physical Phenotype (PP), and Vitality Phenotype (VP) tools with the entire old cohort (21–28 months) represented as parts of a whole (**A**). **B** The cohort is distinctively classified according to each assessment tool, FI, PP, and VP. Each tool is represented by a node. The initial node on the left of the diagram represents the proportion of animals classified as Non-frail or Frail when utilizing the FI tool. The central node represents the proportion of animals from the same cohort classified as Robust, Prefrail, or Frail using the PP tool, while the rightmost node represents the proportion of animals from the same cohort classified as Robust, Prefrail, or Frail when employing the VP (*). Links connecting nodes illustrate how the cohort classification is influenced by each tool. Numbers within nodes represent the sample size classified in each category, whereas numbers within connecting links indicate how many mice changed or maintained their classificatory status between tools. Age distribution is described in Table 1

We observed that the two assessment tools did not identify the female cohort similarly: the FI identified nearly 3 times more female mice as frail compared to the PP (Fig. 1a). Previous findings comparing both tools in male C57BL/6 mice showed that the Frailty Index identified 44.4% of the cohort as frail, whereas the Frailty Phenotype found no mice to be frail and a modified version of the same tool identified only 16.6% of mice as frail [19]. In clinical practice, there is substantial evidence indicating that, at all ages, females tend to exhibit higher levels of frailty compared to males [22–24]. Sex differences in frailty in clinics are influenced by a complex interplay of behavioral, social, and biological factors, although the precise mechanisms remain unclear [25, 26]. Preclinical studies in murine models demonstrate sex-specific variations in frailty assessments depending on the methodologies employed [21, 27]. Inflammation and immune responses, hormonal dysregulation, and genetic factors have all been proposed as mechanisms contributing to sex differences in frailty [25, 28–30]. Further research, particularly in preclinical models, is essential for elucidating these complex sex differences in frailty.

Our finding suggests that these two assessment tools are rather complementary than interchangeable. This suggests that each tool may target distinct frailty markers or pathways and could potentially be more effective if integrated into a new, more comprehensive classification system.

Using a cohort of 190 female mice, the FI classified ~ 77% as Non-frail and ~ 23% as Frail, while the PP identified ~ 78% of animals as Robust (Non-frail), ~ 15% as Prefrail (Non-frail), and ~ 7% as Frail (Fig. 1a). The most primordial difference between both tools is given by the evaluation method, and the Frailty Index is mostly based on the visual inspection of the animal, whereas the PP focuses on performance more than appearance. The second most relevant difference relies on the categorization method. The Frailty Index uses a binary classification, Frail versus Non-frail (positive or negative for frailty), while the PP adds an intermediary frailty state, the Prefrail. The observed reduction of nearly 16% of animals first classified as Frail when using the FI tool and then classified as Robust or Prefrail using the PP suggests that the FI overestimates considerably the frailty status across the cohort. Furthermore, when examining the Sankey diagram, we noted that 25 out of 146 animals (17%) classified as Robust when using the FI were then classified as Prefrail (21) and Frail (4, Fig. 1b) when categorized by the PP. Together, these differences indicate that the absence of an intermediary frailty state in the classification method not only implies the overestimation of Frailty, but also Robustness. Still, it is important to emphasize that not all changes in frailty classification were destined to Prefrail when using the PP. Indeed, 20 out of 44 animals (~ 45%) classified as Frail by the FI were then classified as Robust by the PP, while only 13 were reclassified as Prefrail (Fig. 1b). Interestingly, 45% of animals that accumulated a lot of health deficits scoring poorly with FI performed well physically when examined by the PP, once again suggesting that each tool targets distinct age-related impairments and that not all physiological declines occur simultaneously. Thus, it seems that combining tools to investigate aging decline within an organism might increase the chances of identifying a risk of frailty development.

### Merging Frailty Index and Physical Phenotype, the Vitality Phenotype

Considering that both PP and FI are complementary and not interchangeable tools, we decided to merge both into a more comprehensive one. Since the PP combined multiple physical evaluations, we decided to incorporate the FI (health deficits) as one of the criteria. With a new criterion added to the evaluation, the updated frailty tool will be now referred to as the Vitality Phenotype (VP).

The VP increased the Prefrail classification by nearly 10% as compared to PP due to a reduction of nearly 2% of animals classified under the Frail category and 8% from Robust (Fig. 1a, b).

### Defining cutoff values for Vitality Phenotype criteria

Frailty is a state of vulnerability defined by the loss of reserves due to the accumulative decline of multiple physiological systems. In this context, the VP aims to identify physical signs of frailty by means of an observational and physical performance evaluation. Due to the heterogeneity of the frailty syndrome, the VP encompasses several criteria, each of which accounts as positive or negative according to a given cutoff value. Defining cutoff values is a great challenge not only in clinical practice, but also in animal research. Indeed, in vivo models exhibit significant variability across studies; factors such as genetic background, age, sex, type of bedding, housing conditions, dietary composition, and microbiota can all influence research outcomes [31–33]. This variability often complicates standardization efforts and hinders the reproducibility of findings. We thus suggest that cutoff values should ideally be defined within each research protocol until the field establishes standardization of cutoff values for mice that can be reproduced within each laboratory [9]. In longitudinal studies, tracking individual aging mice is ideal for observing the gradual transition from robustness to frailty [9]. This approach recognizes that frailty is not solely based on age but also on the decline in physiological performance. In contrast, cross-sectional studies only capture a snapshot of differences between groups at a single point in time, rather than tracking changes within the same subjects over a prolonged period. Therefore, when employing a cross-sectional or short-term intervention design, we propose that frailty cutoff values be determined using a reference group. Since the field has not yet established reproducible standard values for frailty-related declines in physical performance, the major selection factor for the reference group is chronological age. For instance, if a cutoff value is set using a very old cohort in a cross-sectional study, a disabled mouse could be incorrectly classified as robust, and conversely, a healthy mouse could be misclassified as frail. Therefore, using an appropriate age as a reference for frailty remains the best approach when working with cross-sectional or short-term studies.

### Defining a reference group for identifying cutoff values for frailty

Despite the enormous advantage regarding shortened lifespans, limited genetic variability, and regulated environmental conditions, defining animal models of frailty is rather challenging, especially concerning its true potential of *reverse translation* from clinical practice. This is also true when choosing the research design and frailty assessment tools. Defining the physiological status/profile that best represents a threshold for a given decline is crucial to determining positive criteria in frailty. Based on a longitudinal approach, our group has previously identified that the reference group for identifying cutoff values should have (1) 100% survival, (2) correspondence to human lifespan: 23 months for a mouse falls within the ~ 65–75 range of human years [34] that corresponds to the initial age brackets assessed by Fried et al. [2], and (3) reversibility potential that would provide adequate time to implement possible life-changing interventions [9]. Herein, we propose that in a cross-sectional study format, the age-driven reference group should be set at an age in which frailty onset is more likely to occur which would still meet 100% survival as well as provide the window for therapeutic interventions, favoring Prefrail classification.

Selecting an appropriate age range where frailty is expected to emerge is crucial for accurately identifying frailty thresholds and defining cutoffs, helping to avoid over- or underestimation in animal classification. For any criterion to be marked as positive, a well-established baseline is essential. In this regard, control groups provide a vital standard for assessing experimental outcomes. While controls are widely recognized in animal models, they are often overlooked in frailty research. Including a specific control for frailty (rather than just aging) helps differentiate frailty-specific findings from those driven by aging alone.

**Fig. 2:**
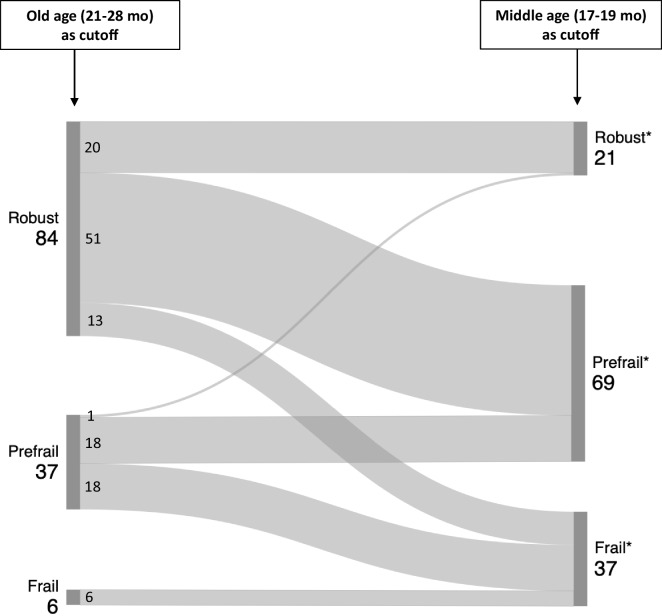
Impact of adding a reference group to define cutoff for frailty classification. Evaluation of the impact of using a reference group (middle age (17–19 months)) to define cutoffs on frailty status within the old cohort. Two nodes represent how the old cohort (21–28 months) is classified when cutoffs are based on two age selections: old age (21–28 months) and middle age (17–19 months*). Within each node, the proportion along with the accurate number of animals classified as Robust, Prefrail, and Frail are illustrated. Links connecting the nodes trace how the cohort classification is influenced by each cutoff. Age distribution is described in Table 2

In a longitudinal study following the prevalence of frailty across the lifespan, our team identified frailty onset in both males and females at 17 months [35, 36]. We thus selected this age to establish an age-matched control group for frailty, which would run parallel to the frail/older cohort in cross-sectional studies. Cutoffs would then be defined by the 17-month-old control group and applied to the groups of interest which would increase the number of animals classified as “Prefrail” or “Frail” consistently (Fig. 2). This age selection would also enable earlier frailty detection, facilitating the identification of prefrail animals—a critical stage for implementing potentially life-changing interventions [35]. Without setting a prefrail cutoff, frailty status may be underestimated, with both Prefrail and Frail animals potentially being misclassified as Robust. Importantly, although we posit that chronological age represents the optimal selection criterion for the reference group in a cross-sectional study, this principle does not extend to the experimental group (old mice). A wide age range was employed for the old group to facilitate a more comprehensive classification of subjects across all three frailty categories. Restricting the selection to a specific age would have significantly reduced the number of animals classified across all frailty categories, thereby compromising the validity of our frailty analysis. Notably, this strategy prioritizes the establishment of consistent Robust, Prefrail, and Frail groups over stringent age selection, as the primary focus is on frailty rather than aging per se. Thus, the experimental groups were defined based on physical performance outcomes, not chronological age, ensuring a more accurate and meaningful assessment of frailty.

### Correlating biological with frailty markers

**Fig. 3:**
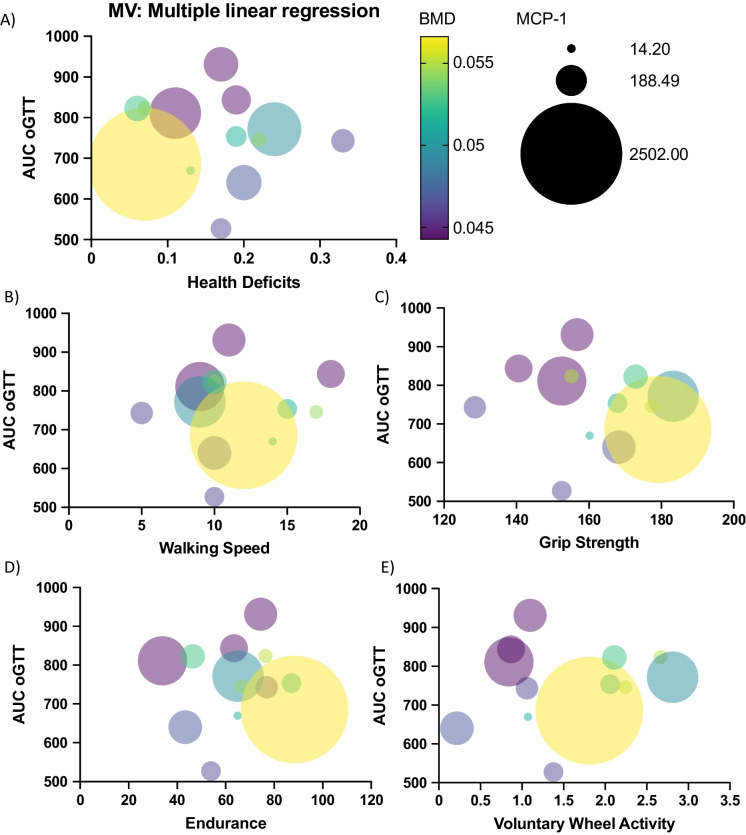
Correlating biological markers with frailty criteria. Correlation between various biological markers and physical frailty criteria. In all panels, Y axes represent AUC oGTT (a.u.), color grading BMD, and circle size MCP-1 levels. Panels are differentiated by X axes that represent each physical frailty criteria: **A** Health Deficits, **B** Walking Speed, **C** Grip Strength, **D** Endurance, and **E** Voluntary Wheel Activity. AUC, area under the curve; BMD, bone mineral density; MCP-1, monocyte chemoattractant protein-1; oGTT, oral glucose tolerance test. Frailty criteria units are described in Table 3

Numerous pathophysiological mechanisms contribute to frailty. The current clinical conceptualization of frailty focuses on three main biological systems: metabolic, stress response, and neuromuscular, which involve endocrine dysregulation, cellular senescence, and chronic inflammation [3]. The Vitality Phenotype includes various criteria that investigate physical performance and assess neuromuscular function, such as the rotarod, treadmill, and grip force test. Moreover, the health deficits criteria include observations such as alopecia, distended abdomen, and rectal prolapse, all deficits that may reflect stress (e.g., chronic inflammation) and/or metabolic dysfunction. Yet, it is not clear whether physical measurements correlate with biological markers of frailty that reflect the decline of the previously mentioned systems. We thus investigated correlations between specific frailty criteria from the Vitality Phenotype and biological markers of glucose homeostasis (e.g., area under the curve from oral glucose tolerance test, AUC oGTT, Fig. 3 and Table 3), bone mineral density (BMD, Fig. 3 and Table 3), inflammation (plasma levels of MCP-1, Fig. 3 and Table 3), and heart rate (HR, shown in Table 3). We observed that the only biological marker that correlated with two frailty criteria was BMD (Fig. 3 and Table 3). BMD inversely correlated with both grip strength and voluntary wheel activity (Fig. 3C and E, respectively, and Table 3). These data suggest that among the analyzed biological markers, BMD is likely the only marker influenced by performance. Both grip force and wheel activity involve increased weight-bearing stress on the animals’ limbs, providing a reasonable explanation as to why animals with worse performance in these criteria had lower whole BMD.

**Table 3:** Correlation values of biological markers and frailty criteria: This table provides the correlation values (*R*^2^) and their statistical significance (*P* values) between various biological markers and frailty criteria. It highlights the significant relationships that can help inform frailty assessments and potential intervention strategies. The table includes correlations for AUC oGTT, BMD, MCP-1, and HR with multiple frailty indicators, providing a comprehensive view of how these biomarkers relate to frailty status. AUC, area under the curve; a.u., arbitrary units; BMD, bone mineral density; HR, heart rate; MCP-1, monocyte chemoattractant protein-1; oGTT, oral glucose tolerance test

Correlation	Health deficits (a.u.)		Walking speed (rpm)		GFT (g)		Distance to exhaustion (m)		Voluntary wheel activity (km/day)	
	*R*^2^	*P* (two-tailed)	*R*^2^	*P* (two-tailed)	*R*^2^	*P* (two-tailed)	*R*^2^	*P* (two-tailed)	*R*^2^	*P* (two-tailed)
AUC oGTT (a.u.)	0.006866	0.7519	0.009814	0.7052	0.00724	0.7454	0.01455	0.6446	0.03849	0.4504
BMD (g/cm^2^)	0.1657	0.1049	0.07541	0.2861	0.2571	0.0378*	0.1417	0.1363	0.3461	0.013*
MCP-1 (pg/ml)	0.1125	0.2626	0.003078	0.8571	0.1461	0.1974	0.09575	0.3036	0.007355	0.7806
HR (bpm)	0.000004129	0.9938	0.0132	0.6606	0.03901	0.4473	0.09638	0.2252	0.05615	0.3598

### Limitations to the study

Given the multi-project nature of this data analysis, our study is subject to certain limitations. One such limitation is the broad mice aging. Although our focus is on biological rather than chronological aging, the reproducibility of our findings may be challenging, which also applies to the research design. Our considerations did not define specific information such as the number of animals from each age range and the age range itself. Another limitation of the present study is the absence of interrater reliability. Due to working with multiple projects over an extended period, the assessments were not performed by the same rater across projects, but only within each project. As we deal with numerous subjective assessments, variability in the rating range may have occurred as a result of this inconsistency.

## Conclusion

This study provides valuable insights into the assessment of frailty in female mice using different tools and approaches. The comparison between the Frailty Index (FI) and Physical Phenotype (PP) revealed significant differences in frailty classification, highlighting the complementary nature of these tools rather than their interchangeability. The newly proposed Vitality Phenotype (VP), which combines elements from both FI and PP, offers a more comprehensive evaluation of frailty status. The study emphasizes the importance of defining appropriate cutoff values for frailty criteria, particularly in cross-sectional studies. The use of a reference group at 17 months of age, where frailty onset is likely to occur, is suggested as an effective approach for establishing these cutoffs, which would allow for better identification of prefrail animals, a critical stage for potential interventions. The investigation of correlations between frailty criteria and biological markers revealed that bone mineral density (BMD) was the only marker significantly associated with multiple frailty criteria. This finding underscores the potential importance of BMD as a biological indicator of frailty in female mice. Overall, this research contributes to the standardization of frailty assessment in animal models and highlights the need for a multifaceted approach to evaluating frailty. The insights gained from this study can inform future research designs and potentially improve the translation of findings from animal models to human clinical applications in the field of aging and frailty.

## References

[1] Dent E, Kowal P, Hoogendijk EO. Frailty measurement in research and clinical practice: a review. Eur J Intern Med. 2016;31:3–10. 27039014 10.1016/j.ejim.2016.03.007

[2] Fried LP, et al. Frailty in older adults: evidence for a phenotype. J Gerontol A Biol Sci Med Sci. 2001;56:M146-156. 11253156 10.1093/gerona/56.3.m146

[3] Perazza LR, Brown-Borg HM, Thompson LV. Physiological systems in promoting frailty. In: Comprehensive Physiology. John Wiley & Sons, Ltd; 2022. pp. 3575–3620.. 10.1002/cphy.c210034. 10.1002/cphy.c210034 PMC9531553 35578945

[4] Walston J, Buta B, Xue Q-L. Frailty screening and interventions: considerations for clinical practice. Clin Geriatr Med. 2018;34:25–38. 29129215 10.1016/j.cger.2017.09.004 PMC5726589

[5] Fried LP, Ferrucci L, Darer J, Williamson JD, Anderson G. Untangling the concepts of disability, frailty, and comorbidity: implications for improved targeting and care. J Gerontol A Biol Sci Med Sci. 2004;59:255–63. 15031310 10.1093/gerona/59.3.m255

[6] Rockwood K, et al. A Frailty Index based on deficit accumulation quantifies mortality risk in humans and in mice. Sci Rep. 2017;7:43068. 28220898 10.1038/srep43068 PMC5318852

[7] Liu H, Graber TG, Ferguson-Stegall L, Thompson LV. Clinically relevant frailty index for mice. J Gerontol A Biol Sci Med Sci. 2014;69:1485–91. 24336799 10.1093/gerona/glt188 PMC4271019

[8] Whitehead JC, et al. A clinical frailty index in aging mice: comparisons with frailty index data in humans. J Gerontol A Biol Sci Med Sci. 2014;69:621–32. 24051346 10.1093/gerona/glt136 PMC4022099

[9] Baumann CW, Kwak D, Thompson LV. Phenotypic frailty assessment in mice: development, discoveries, and experimental considerations. Physiology. 2020;35:405–14. 33052773 10.1152/physiol.00016.2020 PMC7864238

[10] Bautmans I, Gorus E, Njemini R, Mets T. Handgrip performance in relation to self-perceived fatigue, physical functioning and circulating IL-6 in elderly persons without inflammation. BMC Geriatr. 2007;7:5. 17331228 10.1186/1471-2318-7-5 PMC1820598

[11] Ding C, Parameswaran V, Udayan R, Burgess J, Jones G. Circulating levels of inflammatory markers predict change in bone mineral density and resorption in older adults: a longitudinal study. J Clin Endocrinol Metab. 2008;93:1952–8. 18285417 10.1210/jc.2007-2325

[12] Fontana L, et al. Identification of a metabolic signature for multidimensional impairment and mortality risk in hospitalized older patients. Aging Cell. 2013;12:459–66. 23496093 10.1111/acel.12068 PMC6126373

[13] Kilgour AH, et al. Seropositivity for CMV and IL-6 levels are associated with grip strength and muscle size in the elderly. Immun Ageing A. 2013;10:33. 10.1186/1742-4933-10-33 PMC3765201 23938060

[14] Perazza LR, Gower AC, Brown-Borg HM, Pajevic PD, Thompson LV. Protectin DX as a therapeutic strategy against frailty in mice. GeroScience. 2023;45:2601–27. 37059838 10.1007/s11357-023-00789-3 PMC10651819

[15] Lim YJ, et al. Frailty assessment in community-dwelling older adults: a comparison of 3 diagnostic instruments. J Nutr Health Aging. 2020;24:582–90.32510110 10.1007/s12603-020-1396-2

[16] Hwang A-C, et al. Longitudinal changes of frailty in 8 years: comparisons between physical frailty and frailty index. BMC Geriatr. 2021;21:726. 34922488 10.1186/s12877-021-02665-1 PMC8684153

[17] Theou O, Rockwood K. Comparison and clinical applications of the frailty phenotype and Frailty Index approaches. Interdiscip Top Gerontol Geriatr. 2015;41:74–84. 26301981 10.1159/000381166

[18] Imamura K, et al. Comparison of the association between six different frailty scales and clinical events in patients on hemodialysis. Nephrol Dial Transplant. 2023;38:455–62. 35212731 10.1093/ndt/gfac047

[19] Kane AE, et al. A comparison of two mouse frailty assessment tools. J Gerontol A Biol Sci Med Sci. 2017;72:904–9. 28549083 10.1093/gerona/glx009 PMC5861875

[20] Mach J, et al. Preclinical frailty assessments: phenotype and frailty index identify frailty in different mice and are variably affected by chronic medications. Exp Gerontol. 2022;161:111700. 35032570 10.1016/j.exger.2022.111700

[21] Seldeen KL, et al. High intensity interval training improves physical performance in aged female mice: a comparison of mouse frailty assessment tools. Mech Ageing Dev. 2019;180:49–62. 30951786 10.1016/j.mad.2019.04.001 PMC9841971

[22] Theou O, et al. Measuring frailty using self-report and test-based health measures. Age Ageing. 2015;44:471–7. 25687601 10.1093/ageing/afv010 PMC4411224

[23] Dallmeier D, et al. Frailty Index and sex-specific 6-year mortality in community-dwelling older people: the ActiFE study. J Gerontol Ser A. 2019. 10.1093/gerona/glz051. 10.1093/gerona/glz051 30789659

[24] Shi J, et al. Sex differences in the limit to deficit accumulation in late middle-aged and older Chinese people: results from the Beijing longitudinal study of aging. J Gerontol A Biol Sci Med Sci. 2014;69:702–9. 24127426 10.1093/gerona/glt143 PMC4022096

[25] Gordon EH, et al. Sex differences in frailty: a systematic review and meta-analysis. Exp Gerontol. 2017;89:30–40. 28043934 10.1016/j.exger.2016.12.021

[26] Kane AE, Howlett SE. Sex differences in frailty: comparisons between humans and preclinical models. Mech Ageing Dev. 2021;198:111546. 34324923 10.1016/j.mad.2021.111546

[27] Blodgett JM, Theou O, Mitnitski A, Howlett SE, Rockwood K. Associations between a laboratory frailty index and adverse health outcomes across age and sex. AGING Med. 2019;2:11–7. 10.1002/agm2.12055 PMC6880698 31942508

[28] Soysal P, et al. Inflammation and frailty in the elderly: a systematic review and meta-analysis. Ageing Res Rev. 2016;31:1–8. 27592340 10.1016/j.arr.2016.08.006

[29] Samson LD, et al. In-depth immune cellular profiling reveals sex-specific associations with frailty. Immun Ageing. 2020;17:20. 32582361 10.1186/s12979-020-00191-z PMC7310472

[30] Yusipov I, et al. Age-related DNA methylation changes are sex-specific: a comprehensive assessment. Aging. 2020;12:24057–80. 33276343 10.18632/aging.202251 PMC7762479

[31] Jackson SJ, et al. Does age matter? The impact of rodent age on study outcomes. Lab Anim. 2017;51:160–9. 27307423 10.1177/0023677216653984 PMC5367550

[32] Ericsson AC, et al. The influence of caging, bedding, and diet on the composition of the microbiota in different regions of the mouse gut. Sci Rep. 2018;8:4065. 29511208 10.1038/s41598-018-21986-7 PMC5840362

[33] Doetschman T. Influence of genetic background on genetically engineered mouse phenotypes. Methods Mol Biol Clifton NJ. 2009;530:423–33. 10.1007/978-1-59745-471-1_23 PMC2805848 19266333

[34] Flurkey K, Currer JM, Harrison DE. Chapter 20 - Mouse models in aging research. In: Fox JG, et al, editors. The mouse in biomedical research (second edition). Academic Press: Burlington; 2007. pp. 637–672. 10.1016/B978-012369454-6/50074-1.

[35] Baumann CW, Kwak D, Thompson LV. Assessing onset, prevalence and survival in mice using a frailty phenotype. Aging. 2018;10:4042–53. 30562163 10.18632/aging.101692 PMC6326660

[36] Kwak D, Baumann CW, Thompson LV. Identifying characteristics of frailty in female mice using a phenotype assessment tool. J Gerontol A Biol Sci Med Sci. 2020;75:640–6. 30958526 10.1093/gerona/glz092 PMC7328207

